# Combining data discretization and missing value imputation for incomplete medical datasets

**DOI:** 10.1371/journal.pone.0295032

**Published:** 2023-11-30

**Authors:** Min-Wei Huang, Chih-Fong Tsai, Shu-Ching Tsui, Wei-Chao Lin

**Affiliations:** 1 Kaohsiung Municipal Kai-Syuan Psychiatric Hospital, Kaohsiung, Taiwan; 2 Department of Physical Therapy and Graduate Institute of Rehabilitation Science, China Medical University, Taichung, Taiwan; 3 Department of Information Management, National Central University, Taoyuan, Taiwan; 4 Department of Digital Financial Technology, Chang Gung University, Taoyuan, Taiwan; 5 Department of Information Management, Chang Gung University, Taoyuan, Taiwan; 6 Division of Thoracic Surgery, Chang Gung Memorial Hospital at Linkou, Taoyuan, Taiwan; Sunway University, MALAYSIA

## Abstract

Data discretization aims to transform a set of continuous features into discrete features, thus simplifying the representation of information and making it easier to understand, use, and explain. In practice, users can take advantage of the discretization process to improve knowledge discovery and data analysis on medical domain problem datasets containing continuous features. However, certain feature values were frequently missing. Many data-mining algorithms cannot handle incomplete datasets. In this study, we considered the use of both discretization and missing-value imputation to process incomplete medical datasets, examining how the order of discretization and missing-value imputation combined influenced performance. The experimental results were obtained using seven different medical domain problem datasets: two discretizers, including the minimum description length principle (MDLP) and ChiMerge; three imputation methods, including the mean/mode, classification and regression tree (CART), and k-nearest neighbor (KNN) methods; and two classifiers, including support vector machines (SVM) and the C4.5 decision tree. The results show that a better performance can be obtained by first performing discretization followed by imputation, rather than vice versa. Furthermore, the highest classification accuracy rate was achieved by combining ChiMerge and KNN with SVM.

## 1. Introduction

Data pre-processing is an important step in the data mining or knowledge discovery in databases (KDD) process that can affect the final mining result. The aim of data pre-processing is to perform data transformation and cleaning tasks to improve the quality of the data to be analyzed at a later stage [1, 2]. For instance, data discretization can be used to transform continuous variables into discrete data in a collected dataset in which some features (or variables) such as age, income, and financial ratio are recorded as continuous values. Some data-mining algorithms, such as the decision tree, Apriori, and Naive Bayes algorithms, take advantage of data discretization to develop more effective and efficient models [3, 4]. Moreover, discrete attributes are easier to understand, use, and explain [4, 5].

In addition, using well-chosen discretization algorithms (or discretizers) can provide some advantages for most data-mining algorithms, including data reduction and simplification by minimizing information loss during the discretization process, speeding up the learning process, and yielding more accurate, compact, and shorter results. Discrete attributes are easier to understand, use, and explain [4, 5].

In related literature, data discretization has been widely considered to process various medical domain problems, for example, by Oo and Naing [6] for heart disease, diabetes, and hepatitis disorders; Lakshmi and Vadivu [7] for extracting association rules from medical health records; Chern et al. [8] for telehealth service prediction; Alexandre et al. [9] for breast-tissue and yeast datasets; Diamant et al. [10] for respiratory tract infection; and Aristodimou et al. [11] and Kaya and Tekin [12] for various medical domain datasets collected from the UCI Machine Learning Repository, to name a few.

However, whether the collected dataset contains continuous variables or requires data discretization, in practice, some variables will contain missing values because of problems with the database system, network, improper or mistaken data entries, etc. [13]. Many medical datasets suffer from incompleteness, such as microarray gene expression datasets [14, 15], metabolomics data [16], diabetes data [17], clinical electronic health records [18], heart failure data [19], and other biomedical datasets [20].

Unfortunately, without pre-processing, most data-mining algorithms cannot handle incomplete datasets directly. Recently, many techniques have been adapted for missing-value imputation by developing a prediction model to estimate some values to replace the missing ones [13, 21, 22].

The collected datasets for the previously mentioned medical domain problems, such as diabetes data and clinical health records, may contain some continuous variables as well as missing values. In this case, both discretization and missing value imputation steps have to be used to successfully develop effective learning models.

However, this scenario raises an important research issue for determining the best order to combine the two data pre-processing steps, which has never been done before. That is, given a dataset containing some continuous feature variables as well as missing values, if discretization is performed first, the selected continuous feature variables are transformed into discrete ones, whereas the missing values remain unchanged. The second step is to develop the imputation model based on the transformed discrete feature variables to impute the missing values with discrete ones. On the contrary, if missing value imputation is performed first, the imputation model is developed based on the original continuous feature variables to impute the missing values with continuous ones. The second step is to perform data discretization to transform all of the continuous feature variables, including the imputed ones, into discrete ones. Although both combination orders will generate datasets containing discrete feature values, these values will almost certainly differ, which may affect the final mining results. This is because related literatures, such as Tsai and Hu [23] and Lin et al. [24], have shown that different imputation algorithms perform differently on imputing discrete and continuous variables, i.e. different algorithms have their own strengths in imputing specific data type of missing values. In this case, given a training dataset containing continuous variables and missing values, executing the two orders of combining discretization and missing value imputation will generate two different processed datasets since the same imputation algorithms are used to impute discrete variables (i.e. the first combination order) and continuous variables (i.e. the second combination order), respectively.

Therefore, the objective is to examine the effect of the order in which discretization and missing-value imputation are performed on the performance of different classifiers. The contributions of this study are twofold. First, for the research problem of combining both data discretization and missing value imputation, we present two previously unexplored procedures for combining both steps for performance comparison. Second, regarding the experimental results, the best procedure as well as the combination of techniques identified can be regarded as a representative baseline for future research.

The remainder of this paper is organized as follows: Section 2 provides an overview of the literature on data discretization and missing-value imputation. Section 3 describes the research methodology, including the process involving two different combination orders and the experimental setup. Section 4 presents the experimental results, and Section 5 concludes the paper.

## 2. Literature review

### 2.1 Data discretization

The aim of data discretization is to transform a set of continuous variables into discrete variables. In particular, a finite number of intervals with associated categorical values are generated to act as non-overlapping partitions within a continuous domain [5].

The discretization process is defined as follows: Given a dataset *S* consisting of *N* examples, *M* variables (or attributes), and *C* target classes, a discretization algorithm (or discretizer) is used to discretize the continuous variable *A* of *S* into *k* discrete and disjoint intervals $D_{A}=\{{[d}_{0},d_{1}],{[d}_{1},d_{2}],\ldots ,{[d}_{k_{A-1}},d_{k_{A}}]\}$, where *d*_0_ denotes the minimal value, $d_{k_{A}}$ denotes the maximal value, and *d*_*i*_<*d*_*i*+1_ (*i* = 0, 1, …, *k*-1). The discrete result *D*_*A*_ is referred to as a discretization scheme for variable *A* and $P_{A}=\{d_{1},d_{2},\ldots ,d_{k_{A-1}}\}$ is the set of cutoff points for variable *A* in ascending order [25].

In general, the discretization process consists of four steps: sorting the continuous feature values to be discretized, evaluating a cut point for splitting or adjacent intervals for merging, splitting or merging the intervals of continuous feature values based on a defined criterion, and stopping at a certain point [4, 26, 27].

According to related literature reviews [5, 25], existing discretizers can be classified into different categories based on their discretization properties, such as static vs. dynamic, univariate vs. multivariate, supervised vs. unsupervised, splitting vs. merging, global vs. local, direct vs. incremental, evaluation measure, parametric vs. nonparametric, top-down vs. bottom-up, stopping condition, disjoint vs. non-disjoint, and ordinal vs. nominal.

### 2.2 Missing value imputation

In practice, collected medical datasets are often incomplete, and there are some missing values. Three types of missingness mechanisms can cause an incomplete dataset problem: missing completely at random (MCAR), missing at random (MAR), and not missing at random (NMAR) [28]. Regardless of the mechanism causing the problem, missing-value imputation must be performed to complete an incomplete dataset.

Specifically, the missing-value imputation process focuses on constructing a model for the estimation of either continuous or discrete values to replace missing values. Thus, missing-value imputation can be regarded as a pattern-classification process in which a set of observed data without missing values is used as the training set to develop a prediction model. The prediction output (or dependent variable) is based on the missing attributes. Incomplete data with missing attribute values in then inputted as testing data into the trained model to produce a suitable output [21].

According to a recent review [13], imputation techniques can be divided into two types: statistical and machine learning. The most widely used statistical techniques are mean/mode, expectation maximization, and linear/logistic regression, whereas k-nearest neighbor, decision tree, and clustering are machine learning techniques.

### 3. The research methodology

#### 3.1 The first combination order: Discretization and missing value imputation

**Fig 1:**
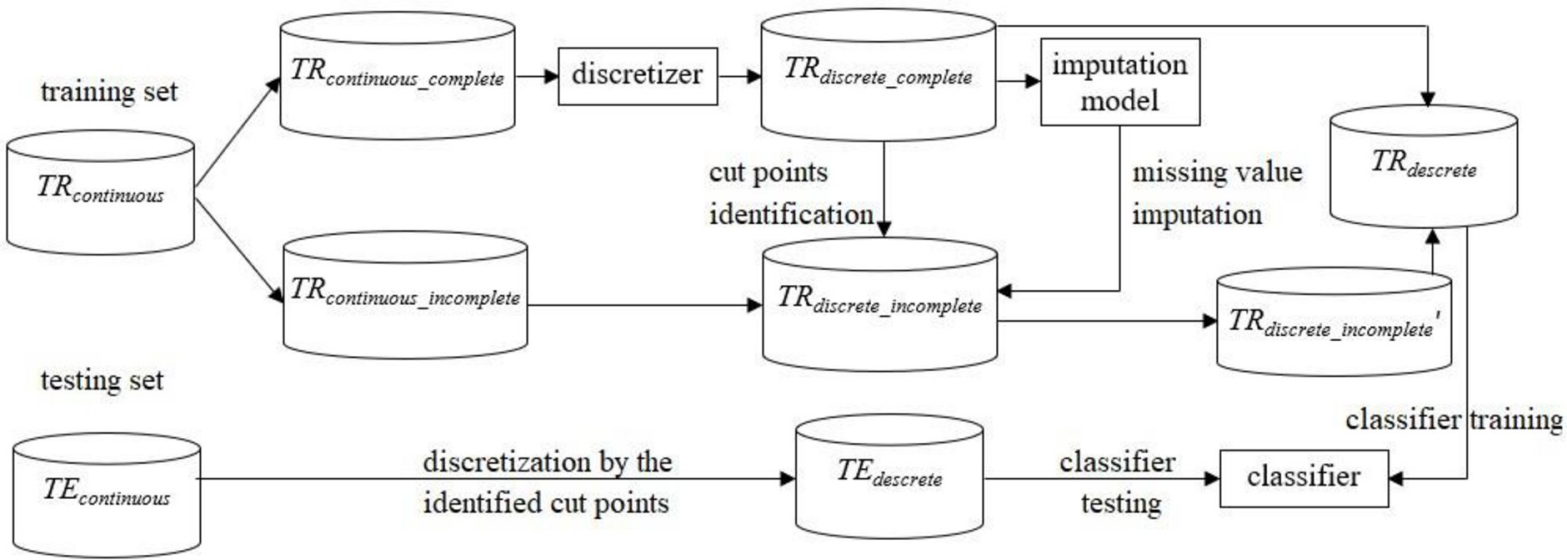
Procedure of first performing discretization and then imputation.

There are two orders in which discretization and missing-value imputation can be combined. Discretization can be performed first, followed by missing-value imputation, or vice versa. The first procedure is shown in Fig 1 and described below.

Dataset *D* is composed of continuous feature variables, which are divided into training and testing sets, denoted as *TR*_*continuous*_ and *TE*_*continuous*_, respectively. In particular, *TR*_*continuous*_ contains missing values. The first step is to divide *TR*_*continuous*_ into complete and incomplete data subsets, denoted as *TR*_*continuous_complete*_ and *TR*_*continuous_incomplete*_, respectively. Following that, the selected discretization algorithm or discretizer is used to transform the continuous feature values of *TR*_*continous_complete*_ into discrete values, denoted as *TR*_*descrete*_*complete*_. Subsequently, the identified cutoff points for different continuous features are used to discretize the continuous feature values of *TR*_*continuous_incomplete*_, except for the missing values, leading to an incomplete subset containing discrete feature values, denoted as *TR*_*discrete*_*incomplete*_.

The selected imputation algorithm is then used to construct an imputation model based on *TR*_*discrete*_*complete*_ to perform missing (discrete) value imputation for *TR*_*discrete*_*incomplete*_. Once *TR*_*discrete*_*incomplete*_ is completely imputed, denoted as *TR*_*discrete*_*incomplete*_′, it is combined with *TR*_*discrete*_*complete*_ to obtain a complete training dataset, denoted as *TR*_*descrete*_. Subsequently, a specific classifier is trained using *TR*_*descrete*_ and the given testing set *TE*_*continuous*_ is discretized based on the related cut points identified by *TR*_*descrete*_*complete*_, denoted as *TE*_*descrete*_. Finally, *TE*_*descrete*_ is used to examine classifier performance.

The following shows the pseudocode of the procedure.

Let *D* = the original dataset composed of continuous feature variables.

Divide *D* into training and testing sets, denoted as *TR*_*continuous*_ and *TE*_*continuous*_, respectively.

Missing value simulation is performed over *TR*_*continuous*_ (c.f. Section 3.3.1).

Divide *TR*_*continuous*_ into complete and incomplete data subsets, denoted as *TR*_*continuous_complete*_ and *TR*_*continuous_incomplete*_, respectively.

Perform data discretization over *TR*_*continuous_complete*_ to produce *D*_*descrete_complete*_ (cutoff points are also identified).

Perform data discretization over *TR*_*continuous_incomplete*_ based on the identified cutoff points by Step 5 to produce *TR*_*discrete_incomplete*_.

Construct missing value imputation model based on *TR*_*discrete_complete*_.

For *i* from 1 to the size of *TR*_*discrete_incomplete*_

   Construct missing value imputation model based on ${TR}_{discrete_{complete}}$

   Perform missing value imputation for *i*

End for

*TR*_*discrete_incomplete*_′ ← all missing data of *TR*_*discrete_incomplete*_ are imputed

Combine *TR*_*descrete_complete*_ and *TR*_*discrete_incomplete*_′ to produce a complete training dataset, denoted as *TR*_*discrete*_.

Train a classifier based on *TR*_*discrete*_.

Perform data discretization over *TE*_*continuous*_ based on the identified cutoff points by Step 5 to produce *TE*_*discrete*_.

Test the classifier based on *TE*_*discrete*_.

**Algorithm 1** Pseudocode for first performing discretization and then imputation

### 3.2 The second combination order: Missing value imputation + discretization

**Fig 2:**
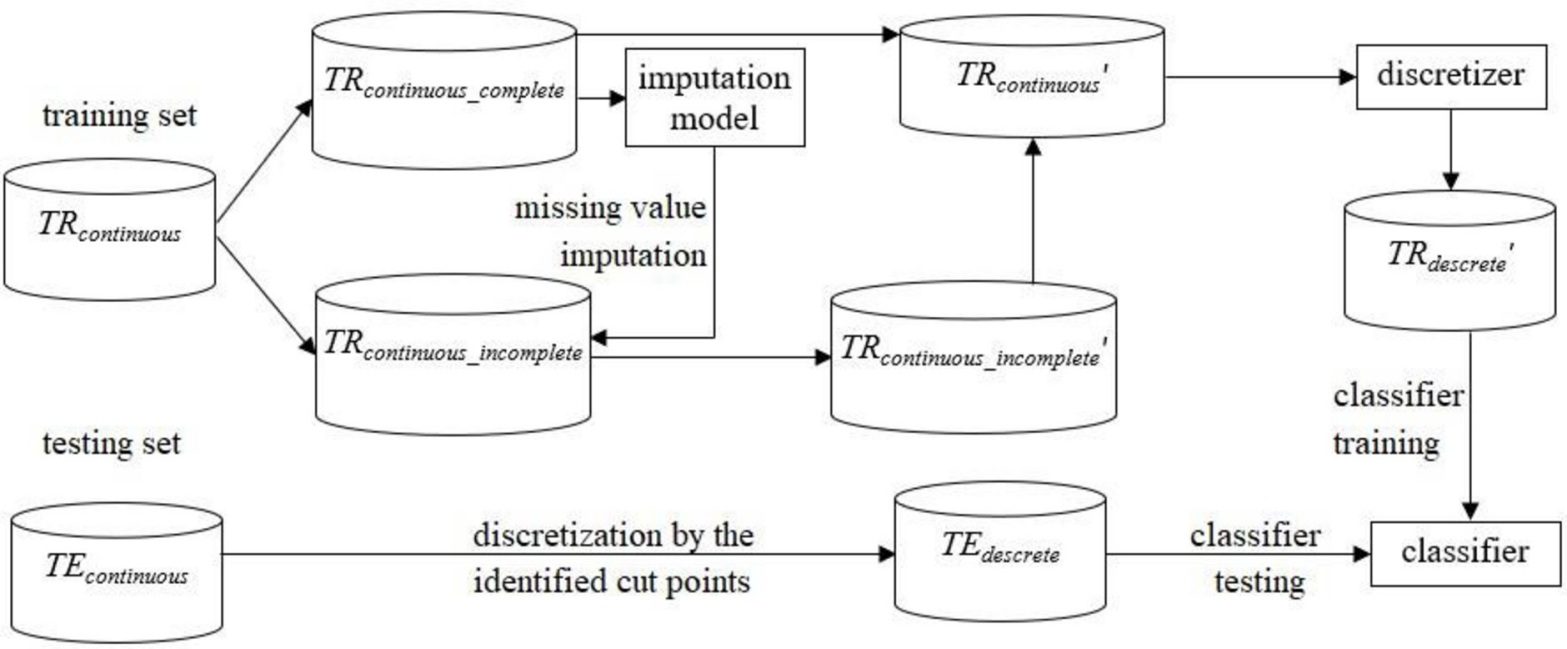
Procedure of first performing imputation and then discretization.

In the second combination, first missing-value imputation is performed, followed by discretization (c.f. Fig 2). First, the selected imputation algorithm is used to construct an imputation model based on *TR*_*continuous_complete*_. The imputation model is then used to impute the continuous feature values for *TR*_*continuous_incomplete*_. Consequently, *TR*_*continuous_incomplete*_ becomes complete and is denoted as *TR*_*continuous_incomplete*_′. Subsequently, the imputed subset *TR*_*continuous_incomplete*_′ is combined with *TR*_*continuous_complete*_ to obtain a complete training dataset, denoted as *TR*_*continuous*_′.

Following that, the chosen discretizer is used to transform all the continuous features of *TR*_*continuous*_′ into discrete features, denoted as *TR*_*discrete*_′. A specific classifier is then trained by *TR*_*discrete*_′ and the testing set *TE*_*continuous*_ is discretized by the identified cut-points using *TR*_*discrete*_′, denoted as *TE*_*descrete*_. Notably, the sets of *TE*_*descrete*_ produced by the two combination orders are not necessarily similar. Finally, *TE*_*descrete*_ is used to examine classifier performance. Therefore, the classifiers trained using *TR*_*discrete*_ produced by the first combination order and *TR*_*discrete*_′ produced by the second combination order are expected to perform differently.

The following shows the pseudocode of the procedure.

Let *D* = the original dataset composed of continuous feature variables.

Divide *D* into training and testing sets, denoted as *TR*_*continuous*_ and *TE*_*continuous*_, respectively.

Missing value simulation is performed over *TR*_*continuous*_ (c.f. Section 3.3.1).

Divide *TR*_*continuous*_ into complete and incomplete data subsets, denoted as *TR*_*continuous_complete*_ and *TR*_*continuous_incomplete*_, respectively.

For *i* from 1 to the size of *TR*_*continuous_incomplete*_

   Construct missing value imputation model based on *TR*_*continuous_complete*_

   Perform missing value imputation for *i*

End for

*TR*_*continuous_incomplete*_′ ← all missing data of *TR*_*continuous_incomplete*_ are imputed

Combine *TR*_*continuous_complete*_ and *TR*_*continuous_incomplete*_′ to produce a complete training dataset, denoted as *TR*_*continuous*_′.

Perform data discretization over *TR*_*continuous*_′ to produce *TR*_*discrete*_′ (cut points are also identified).

Train a classifier based on *TR*_*discrete*_′.

Perform data discretization over *TR*_*continuous*_ based on the identified cut points by Step 8 to produce *TE*_*descrete*_.

Test the classifier based on *TE*_*descrete*_.

**Algorithm 2** Pseudocode for performing imputation first and discretization second

### 3.3 Experimental setup

#### 3.3.1 The datasets

Because the research objective is to assess the performance of the two combination orders for medical domain problem datasets, related medical datasets are selected from the UCI Machine Learning Repository (https://archive.ics.uci.edu/datasets). The major selection criterion is based on the dataset that contains continuous feature variables to perform the data discretization task. In particular, only the datasets that contain more than half of the continuous feature variables are selected. Consequently, seven datasets are chosen, which cover the problems regarding the prediction of diabetes, cancer, heart disease, and Parkinson’s disease, as well as data related to the bioconcentration factor and electroencephalography measurement. The basic information on the seven datasets is listed in Table 1.

**Table 1:** Dataset information.

	No. of features	No. of data samples	No. of classes
Breast cancer (original)	10	699	2
Breast cancer (diagnostic)	32	569	2
EEG eye state	15	14980	2
Parkinson’s	23	197	2
Pima	8	768	2
QSAR	41	1055	2
Statlog	13	270	2

Each dataset is divided into 80% training and 20% testing sets using the 5-fold cross validation method. In addition, each training set is simulated using 10%, 20%, 30%, 40%, and 50% missing rates based on the missing complete at random (MCAR) missingness mechanism. To avoid producing biased imputation results, each missing rate simulation is performed ten times, generating ten different incomplete training sets for each missing rate. The final performance of the classifiers is based on the average of the ten imputation results.

#### 3.3.2 The discretizers

To select the candidate discretizers for this study, related literature comparing various discretizers has shown that supervised discretization methods usually perform better than unsupervised ones [5, 29]. Moreover, recent comparative studies focusing on the data discretization task employed the MDLP and ChiMerge discretizers [24, 30]. This is because Garcia et al. [5], who compared thirty different discretizers, identified that MDLP and ChiMerge both performed reasonably well. With the MDLP, a satisfactory tradeoff between the number of intervals produced and accuracy can be obtained, whereas ChiMerge offers excellent performance for all types of classifiers.

The MDLP can be categorized as a static, univariate, supervised, splitting, local, and incremental method [31]. Potential cutoff points are formed at the boundaries between classes after the continuous feature values are sorted. Specifically, the entropy criterion with minimum description length is used as the stopping rule for selecting useful cutoff points.

However, ChiMerge [32] can be categorized as a static, univariate, supervised, merging, global, and incremental method. It uses the chi-square statistic to discretize numeric attributes by checking each pair of adjacent rows to determine whether the class frequencies of the two intervals are significantly different.

#### 3.3.3 The imputation techniques

For the candidate imputation techniques, mean/mode, CART, and KNN are chosen. This is because, based on the survey conducted by Lin and Tsai [13], they were the most widely used missing value imputation techniques. Moreover, Tsai and Hu [23], who compared six statistical and machine learning-based imputation techniques over 33 datasets, concluded that CART was the better choice for missing value imputation because its imputation result enabled different classifiers to perform reasonably well and it could generate the lowest RMSE (root-mean-square error) for numerical datasets.

The mean and mode were used to impute the continuous and discrete feature values, respectively. In contrast, KNN is a nonparametric method for classification and regression. Given a set of training examples, that is, complete training data without missing values, the output of a given testing dataset (i.e., the missing value to be imputed) is based on the class of its *k*-nearest neighbors for discrete feature values or the average of the values of its *k*-nearest neighbors for continuous feature values.

#### 3.3.4 The classifiers

The performance obtained by combining discretization and missing-value imputation in different orders were examined. Two commonly used classifiers were constructed. The first was the SVM based on the RBF kernel function. SVM has been regarded as one of the core supervised learning techniques [33–35], and it has been widely used as the major technique for various medical domain problems [36–42].

The second is the C4.5 decision tree. Different from SVM, which is a black box algorithm, C4.5 is a white box algorithm that can extract decision rules for different classification tasks. It is not only regarded as one of the top data mining algorithms [35, 43, 44], but also widely employed for medical decision-making [45–49].

Three different classifier training and testing strategies were considered for each dataset to obtain baselines for performance comparison.

- Baseline 1: The classifiers were trained and tested purely on the original dataset with continuous feature values. In other words, discretization and missing-value simulations were not performed.
- Baseline 2: Discretization was performed over the training set without simulations of missing values. Subsequently, training sets with discrete feature values produced by the MDLP and ChiMerge were used to train the classifiers, and the identified cutoff points were used to discretize the testing set to test the trained classifiers.
- Baseline 3: Missing values in the training set were simulated, and the imputed training sets obtained based on the mean, CART, and KNN imputations for continuous feature values were used to train the classifiers.

## 4. Experimental results

### 4.1 Baseline 1 vs. baseline 2

Table 2 lists the classification accuracies of SVM and C4.5 for baselines 1 and 2. In other words, this comparison aimed to determine whether discretization could enhance the performance of the classifiers. The best results obtained for each dataset are underlined.

**Table 2:** Classification accuracies of SVM and C4.5 for baselines 1 and 2.

Dataset	Baseline 1	Baseline 2	
		*ChiMerge*	*MDLP*
*SVM*			
Breast cancer (o)	0.981	0.982	0.977
Breast cancer (d)	0.912	0.961	0.970
EEG eye state	0.543	0.874	0.770
Parkinson’s	0.795	0.877	0.877
Pima	0.765	0.747	0.758
QSAR	0.671	0.694	0.632
Statlog	0.718	0.669	0.673
Avg.	0.769	0.829	0.808
*C4*.*5*			
Breast cancer (o)	0.948	0.950	0.953
Breast cancer (d)	0.902	0.916	0.925
EEG eye state	0.831	0.840	0.805
Parkinson’s	0.887	0.805	0.867
Pima	0.692	0.688	0.752
QSAR	0.611	0.588	0.588
Statlog	0.604	0.587	0.682
Avg	0.782	0.768	0.796

As can be observed, in most cases (i.e., datasets), better SVM classifier performance was obtained when discretization was performed rather than when trained by the original datasets (i.e., baseline 1), regardless of which discretization algorithm was used, the exception being the Pima and Statlog datasets. Specifically, when ChiMerge was used to discretize the continuous feature values, the SVM provided higher rates of classification accuracy than when MDLP was used. However, for the C4.5 classifier, the performance only improved after discretization using MDLP.

These results indicate that discretization allowed classifiers to provide better classification accuracy than without it. However, the discretization algorithm must be carefully selected for some classifiers.

The best performance was obtained using ChiMerge for discretization and SVM for classification. This combination performed best among all selected medical datasets, except for the breast cancer (d), Pima, and Statlog datasets. The best approaches for the former two datasets were MDLP + SVM and MDLP + C4.5, respectively, and the baseline 1 method for the third dataset.

### 4.2 Baseline 1 vs. baseline 3

**Fig 3:**
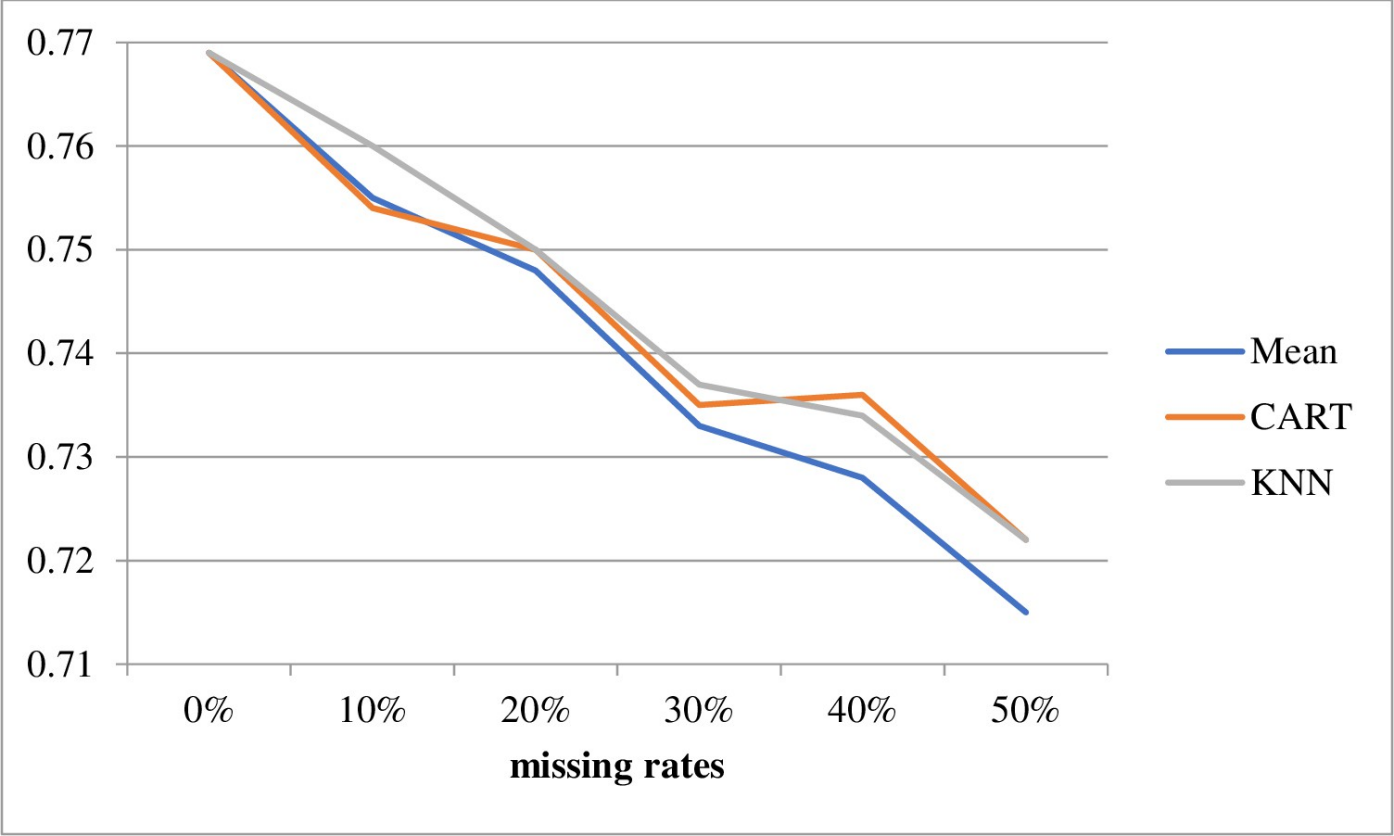
SVM classification accuracies using different imputation methods.

**Fig 4:**
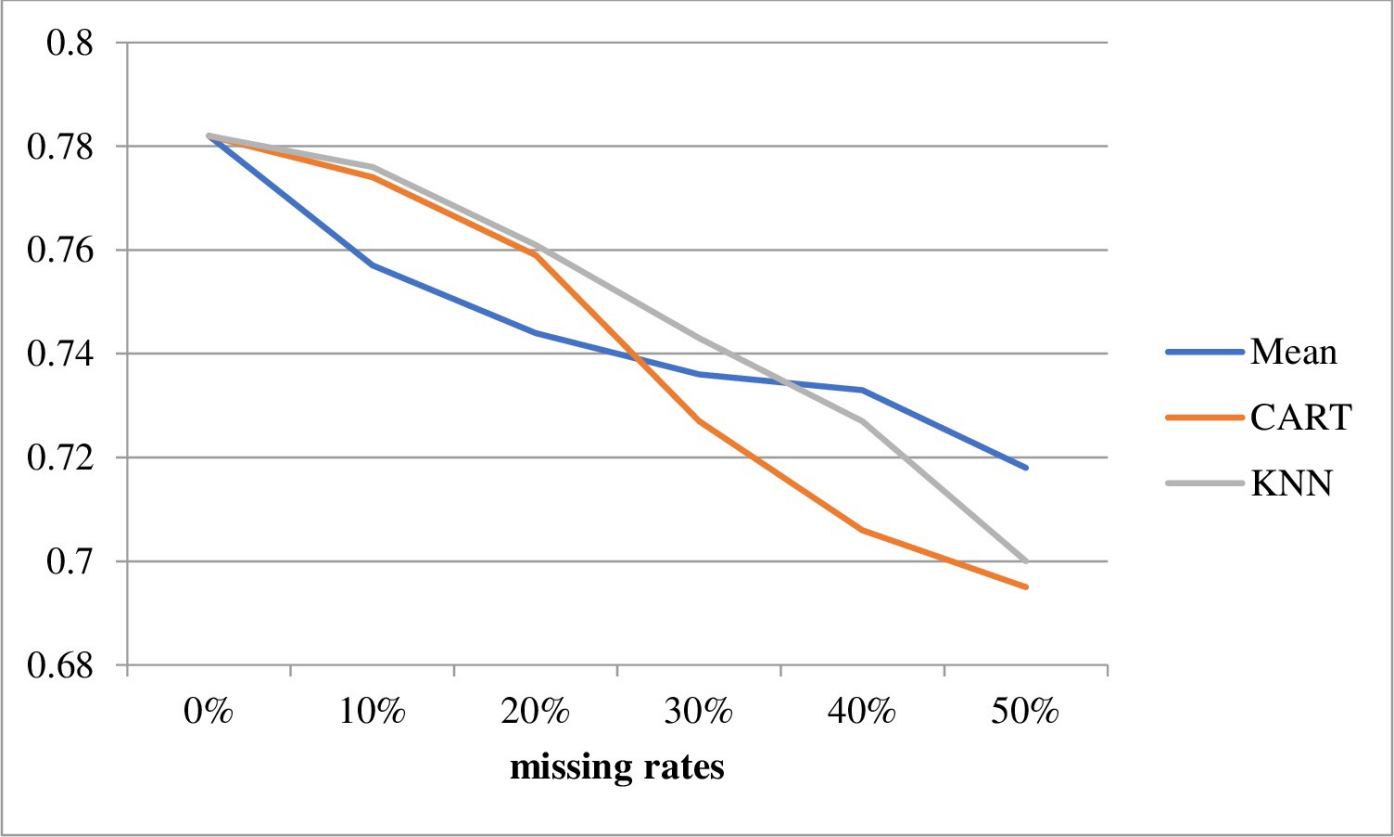
C4.5 classification accuracies using different imputation methods.

The second comparison examined the differences in performance obtained for incomplete datasets with different missing rates. Figs 3 and 4 show the average classification accuracies of SVM and C4.5, respectively, over seven datasets with different missing rates obtained using the mean, CART, and KNN imputation methods. The classification accuracy for the 0% missing rate represented the performance of baseline 1 (0.769).

These results showed that when the missing rates increased, the classification accuracies of the SVM and C4.5 gradually decreased. When the missing rate was lower than 30%, the KNN imputation method outperformed the mean and CART methods, regardless of the classifier used. However, when the missing rate was higher than 30%, CART and KNN performed similarly to the SVM classifier, whereas the mean imputation method outperformed CART and KNN with the C4.5 classifier.

In summary, the best combinations for the imputation methods and classifiers under the 10%, 20%, 30%, 40%, and 50% missing rates were KNN + C4.5, KNN + C4.5, KNN + C4.5, CART + SVM, and KNN + SVM, respectively.

### 4.3 Discretization and missing value imputation vs. missing value imputation and discretization

Table 3 lists the average SVM and C4.5 classification accuracies obtained using the two combination approaches. The results for the seven datasets are numbered from 1 to 7. Each missing rate produces one classification result. The results reported here are based on the average of five classification results, corresponding to missing rates ranging from 10–50%. The best results for each dataset were highlighted.

**Table 3:** Classification accuracies of SVM and C4.5 using different approaches.

	Base-Line 1	discretization + missing value imputation						Missing value imputation + discretization					
		*ChiMerge*			*MDLP*			*mean*	*CART*	*KNN*	*mean*	*CART*	*KNN*
		mode	CART	KNN	mode	CART	KNN	ChiMerge			MDLP		
*SVM*													
1	0.981	0.963	0.959	0.968	0.963	0.960	0.965	0.969	0.959	0.963	0.967	0.960	0.964
2	0.912	0.932	0.939	0.949	0.946	0.948	0.955	0.944	0.915	0.937	0.953	0.947	0.942
3	0.543	0.732	0.742	0.763	0.690	0.694	0.698	0.713	0.743	0.761	0.679	0.695	0.704
4	0.795	0.801	0.837	0.826	0.829	0.831	0.845	0.785	0.815	0.818	0.843	0.830	0.819
5	0.765	0.692	0.699	0.701	0.716	0.705	0.720	0.701	0.688	0.692	0.711	0.710	0.713
6	0.671	0.758	0.769	0.769	0.746	0.720	0.740	0.637	0.630	0.632	0.611	0.604	0.612
7	0.718	0.669	0.662	0.670	0.637	0.641	0.652	0.670	0.668	0.665	0.667	0.646	0.654
Avg.	0.769	0.792	0.801	0.807	0.790	0.786	0.796	0.774	0.774	0.781	0.776	0.770	0.773
Avg.		0.795						0.775					
*C4*.*5*													
1	0.948	0.931	0.919	0.932	0.933	0.919	0.934	0.924	0.916	0.912	0.933	0.922	0.928
2	0.902	0.889	0.892	0.915	0.907	0.909	0.920	0.891	0.897	0.887	0.902	0.904	0.910
3	0.831	0.682	0.674	0.698	0.687	0.664	0.650	0.674	0.680	0.704	0.675	0.679	0.704
4	0.887	0.676	0.689	0.715	0.794	0.787	0.800	0.487	0.543	0.684	0.789	0.777	0.786
5	0.692	0.643	0.630	0.646	0.704	0.674	0.675	0.657	0.623	0.643	0.700	0.682	0.706
6	0.611	0.591	0.596	0.595	0.623	0.633	0.624	0.614	0.582	0.598	0.630	0.623	0.622
7	0.604	0.608	0.582	0.593	0.626	0.621	0.599	0.602	0.593	0.612	0.664	0.645	0.658
Avg.	0.782	0.717	0.712	0.728	0.753	0.744	0.743	0.693	0.691	0.720	0.756	0.747	0.759
Avg.		0.733						0.728					

Regardless of the order in which discretization and missing-value imputation are performed and which algorithms are used, the SVM classifier combinations all perform better than baseline 1. The top three combinations were ChiMerge + KNN, ChiMerge + CART, and MDLP + KNN. Based on the Wilcoxon rank-sum test, these approaches offered significantly better performance than the other approaches (*p*<0.05). However, the baseline performance was significantly better than any of the C4.5 classifier combinations (*p*<0.05). These results showed that the choice of classifier was a key factor affecting the final classification performance obtained with discretization and missing-value imputation combinations.

A comparison between the two combination orders showed that, on average, higher rates of classification accuracy were provided by first performing discretization, followed by missing value imputation, for both SVM and C4.5, than by first performing missing value imputation, followed by discretization, that is, 0.795 vs. 0.775 and 0.733 vs. 0.728 for SVM and C4.5, respectively. However, when only the SVM was used, there was a significant difference in the level of performance between the two combination orders (*p*<0.05).

This result indicates that the problem of imputing continuous feature variables for medical datasets is more difficult than discrete ones. In other words, performing the data discretization process first can simplify the original continuous feature variables, allowing the imputation methods to produce better results, i.e. discrete values, for constructing more effective classifiers. On the contrary, performing missing value imputation first for continuous variables increases the computational complexity of the imputation models and the combined original and imputed continuous variables cannot make the discretizers to produce better discrete values for the latter classifiers.

Table 4 compares the average classification accuracies for SVM and C4.5, obtained using the best algorithm combinations and baselines 2 and 3. Note that in baseline 2, the best results for SVM and C4.5 were based on ChiMerge and MDLP, respectively (cf. Table 2). At baseline 3, the averages of the five classification results corresponding to 10–50% missing rates obtained using the mean, CART, and KNN were compared, and the best imputation method was presented.

**Table 4:** Average classification accuracies of SVM and C4.5.

	Baseline 2	Baseline 3	The best combination algorithm
**SVM**	0.829 (ChiMerge)	0.741 (KNN)	0.807 (ChiMerge + KNN)
**C4.5**	0.796 (MDLP)	0.741 (KNN)	0.759 (KNN + MDLP)

In baseline 3, which represents the results of performing missing value imputation over the datasets containing 10–50% missing rates, the best imputation method for the SVM and C4.5 classifiers was KNN, with an average classification accuracy of 0.741. Combining discretization and missing value imputation, the best algorithm combinations for SVM and C4.5 were ChiMerge + KNN and KNN + MDLP, respectively. The results indicated that, for datasets composed of several numerical features where some values were missing, in addition to performing missing-value imputation, it was better to consider data discretization.

Specifically, for the SVM, first performing discretization with ChiMerge followed by missing value imputation with KNN outperformed the other combination orders and other algorithm combinations. Furthermore, to examine the performance differences between the best combination algorithms by SVM and C4.5 and their corresponding baseline 2, SVM by ChiMerge + KNN performed better than C4.5 by KNN + MDLP, because their performance differences from baseline 2 were 0.022 and 0.037, respectively.

One possible reason ChiMerge can produce better discretization results than MDLP for the latter imputation step is the size of the chosen datasets, including the feature dimensions and numbers of data samples. That is, because ChiMerge treats all of the individual variables as different intervals and repeats the process of merging and sorting intervals from bottom to top, this implies that the chosen datasets were relatively ‘easy’ for ChiMerge to produce better results. Moreover, based on the better discretization results, that is, discrete values, the KNN imputation model was easier and more effective to measure the distances between the observed and missing data.

On the other hand, for C4.5, although the best performance is obtained by performing missing value imputation first by KNN and data discretization second by MDLP, the opposite combination order based on performing data discretization first by MDLP and missing value imputation second by the mode method allows C4.5 to produce very similar accurate rate, i.e. 0.753. Moreover, regarding Table 3, the average performances of the two combination orders show that performing data discretization first and missing value imputation second make both SVM and C4.5 perform better than the ones by the opposite combination order. This provides a general guideline for the order of combining data discretization and missing value imputation.

Among the various algorithm combinations, ChiMerge + KNN was identified to significantly outperform the other algorithm combinations, that is, 0.807 vs. 0.759 (*p* < 0.05). Moreover, ChiMerge + KNN performed better than baseline 2 by > 6.6%, which was higher than that of KNN + MDLP, which performed better than baseline 2 by > 1.8%.

Compared to baseline 1, where discretization is performed over datasets (without missing values), a better algorithm combination can be identified by examining the performance difference between them and their corresponding baseline 1. In other words, a smaller difference indicated that the combined algorithms performed better. Consequently, ChiMerge + KNN was the better choice, with a much smaller difference in performance from baseline 1, much smaller than that for KNN + MDLP, that is, 2.2% (0.829–0.807) vs. 3.7% (0.796–0.759). However, the classifier must be carefully chosen to maximize the final classification performance after discretization and missing-value imputation.

## 5. Conclusion

Data discretization and missing value imputation are two important data pre-processing steps in data mining and analysis: the former focuses on transforming continuous features into discrete ones, and the latter focuses on the estimation of some values to replace the missing ones. In this study, we focused on the problems of processing medical domain datasets that require both discretization and missing-value imputation.

When discretization was performed first, the imputation algorithms were forced to estimate the discrete values of the missing values. By contrast, when imputation was performed first, the algorithms produced continuous values to replace missing values in the later discretization step. The performance obtained using these two combination orders was examined by employing two discretizers, including the minimum description length principle (MDLP) and ChiMerge; three imputation methods, including mean/mode, CART, and KNN; and two classifiers, including SVM and C4.5 decision trees.

Experimental results based on seven different medical domain problem datasets showed that performing discretization first, followed by imputation offered better performance than the other methods for both the SVM and C4.5 classifiers. However, only the SVM with a combination of discretization and imputation provided the closest performance to the SVM with discretization (i.e., baseline 2). These results indicated that the classification technique must be carefully chosen because it could affect the final result after combining discretization and imputation. Specifically, discretization using ChiMerge and imputation using KNN outperformed the other combined algorithms.

However, several issues should be addressed in future studies. First, for medical domain datasets, feature selection plays an important role in enabling classifiers to perform better than those without feature selection [50]. Thus, the effect of feature selection on the combined algorithms is worth investigating. In other words, performing feature selection may highly affect some specific datasets, such as the Pima and Statlog datasets, which may allow baseline 2 to outperform baseline 1. Second, because several medical domain datasets are class-imbalanced, with the number of data samples in one class being much smaller than that in the other, under- and oversampling approaches can be used to balance the datasets to construct more effective classifiers [51]. In this case, it is useful to examine whether sampling approaches can further improve the performance of the combined algorithms. Third, although the chosen discretizers, imputation methods, and classification techniques in this study are well known and widely used for various data mining and medical domain problems, other competitive algorithms can be considered for performance comparison. For the example of classification techniques, classifier ensembles, which are based on combining multiple classifiers, appear to outperform single classifiers [52]. It is worth further examining the difference in performance between classifier ensembles and single classifiers after combining discretization and missing value imputation. Fourth, although many medical domain problem datasets belong to the two-class classification problem, multi-class datasets can also be used for further performance comparison. This is because most multi-class datasets are not only class imbalanced but also more challenging to effectively handle than two-class datasets.
